# Proteo-Transcriptomic Analysis Identifies Potential Novel Toxins Secreted by the Predatory, Prey-Piercing Ribbon Worm *Amphiporus lactifloreus*

**DOI:** 10.3390/md18080407

**Published:** 2020-08-01

**Authors:** Björn Marcus von Reumont, Tim Lüddecke, Thomas Timm, Günter Lochnit, Andreas Vilcinskas, Jörn von Döhren, Maria A. Nilsson

**Affiliations:** 1 Institute for Insect Biotechnology, Justus-Liebig-Universität Gießen, Heinrich Buff Ring 26–32, 35392 Gießen, Germany; andreas.vilcinskas@ime.fraunhofer.de; 2 LOEWE Centre for Translational Biodiversity Genomics (LOEWE-TBG), Senckenberganlage 25, 60325 Frankfurt, Germany; Tim.Lueddecke@ime.fraunhofer.de (T.L.); maria.nilsson-janke@senckenberg.de (M.A.N.); 3 Branch Bioressources, Department Animal Venomics, Fraunhofer Institute for Molecular Biology and Applied Ecology, Ohlebergsweg 12, 35392 Giessen, Germany; 4 Protein Analytics, Institute of Biochemistry, Justus-Liebig-Universität Gießen, Friedrichstrasse 24, 35392 Gießen, Germany; thomas.timm@biochemie.med.uni-giessen.de (T.T.); guenter.lochnit@biochemie.med.uni-giessen.de (G.L.); 5 Institute for Evolutionary Biology and Ecology, Rheinische Friedrich-Wilhelms-Universität Bonn, An der Immenburg 1, 53121 Bonn, Germany; jdoehren@evolution.uni-bonn.de; 6 Biodiversity and Climate Research Centre, Senckenberg Gesellschaft für Naturforschung, Senckenberganlage 25, 60325 Frankfurt am Main, Germany

**Keywords:** nemertean toxins, transcriptomics, proteomics, inhibitor cystine knot/knottin, actitoxins, plancitoxins, nemertotoxins, hoplonemerteans

## Abstract

Nemerteans (ribbon worms) employ toxins to subdue their prey, but research thus far has focused on the small-molecule components of mucus secretions and few protein toxins have been characterized. We carried out a preliminary proteotranscriptomic analysis of putative toxins produced by the hoplonemertean *Amphiporus lactifloreus* (Hoplonemertea, Amphiporidae). No variants were found of known nemertean-specific toxin proteins (neurotoxins, cytotoxins, parbolysins or nemertides) but several toxin-like transcripts were discovered, expressed strongly in the proboscis, including putative metalloproteinases and sequences resembling sea anemone actitoxins, crown-of-thorn sea star plancitoxins, and multiple classes of inhibitor cystine knot/knottin family proteins. Some of these products were also directly identified in the mucus proteome, supporting their preliminary identification as secreted toxin components. Two new nemertean-typical toxin candidates could be described and were named U-nemertotoxin-1 and U-nemertotoxin-2. Our findings provide insight into the largely overlooked venom system of nemerteans and support a hypothesis in which the nemertean proboscis evolved in several steps from a flesh-melting organ in scavenging nemerteans to a flesh-melting and toxin-secreting venom apparatus in hunting hoplonemerteans.

## 1. Introduction

**Figure 1:**
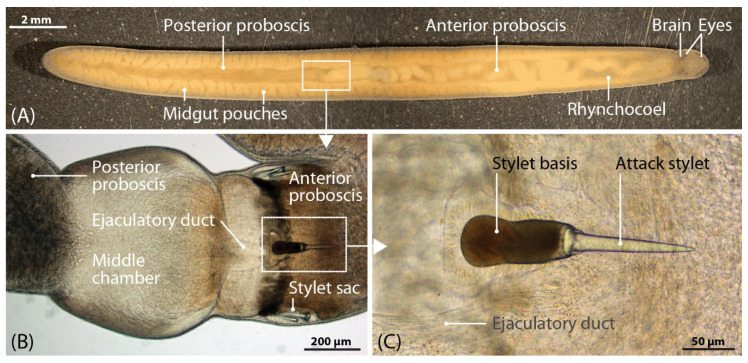
Habitus of *Amphiporus lactifloreus* (**A**) with a magnified stylet apparatus (**B**,**C**).

Nemertea is a phylum of unsegmented worms (ribbon worms) featuring 1300 mostly marine species. It belongs to the disputed superphylum Lophotrochozoa, which contains one third of all marine animals, including sensu stricto polychaetes, mollusks, brachiopods and phoronids [1,2,3]. A recently-proposed system has established three nemertean classes: Paleonemertea, Pilidiophora and Hoplonemertea [4]. All three feature an eversible proboscis, but hoplonemerteans uniquely possess a hardened needle-like proboscis tip that can pierce the prey cuticle, as shown for the topic of this study: *Amphiporus lactifloreus* (Figure 1). Hoplonemerteans probably use the proboscis tip to facilitate the injection of venom during fast strikes, leading to full paralysis within a few minutes [5]. Having attacked and retreated, the predator later returns to its immobilized prey in order to feed. Nemerteans are typically carnivorous predators or scavengers [5] and several species hunt well-defended and, in some cases, venomous species, such as polychaetes, although their ecology and feeding habits are not yet understood in detail [6,7,8,9]. Nemerteans can therefore be included along with cnidarians [10,11], echinoderms and polychaetes [12,13,14] as another ancient marine lineage that produces neurotoxins for defense and/or predation.

Nemertean toxins were first discovered in the epidermal mucus [2] and research has focused on small-molecule components, such as tetrodotoxin and other alkaloids. Few peptide or protein components have been isolated, expressed and characterized in terms of structure and activity [2]. Comprehensive sequence, structural and pharmacological data are only available for a handful of proteins from three species in the class Pilidiophora: *Cerebratulus lacteus* (cytotoxin A-III and neurotoxins B-II and B-IV), *Parborlasia corrugatus* (parbolysin), and *Lineus longissimus* (nemertides α-1, α-2 and β-1). These toxins have been discussed in a comprehensive review [2].

The limited understanding of nemertean toxins in part reflects the absence of a centralized venom system with a distinct venom gland and venom duct that can be milked or dissected easily. Instead, glandular cells that are thought to secrete toxins are distributed throughout the proboscis [15]. Furthermore, nemerteans produce a characteristic mucus layer (as with many marine organisms), and this also contains toxins, which are probably delivered passively as a poison or toxungen [16,17]. These mucosal toxins may be utilized simultaneously for defense and predation without a clear separation of roles.

Few studies have attempted to mine genome or transcriptome data to identify known nemertean toxins and potential novel variants. A whole-animal transcriptomic toxin-profiling study of nine nemertean species revealed several transcripts that match known toxin families from other venomous animals, especially sequences similar to plancitoxin from the crown-of-thorns sea star [18]. These toxins were identified in all nine species, representing all three nemertean classes: Pilidiophora (*Cerebratulus marginatus*, *Lineus lacteus*, *L. longissimus*, and *Lineus ruber*), Palaeonemertea (*Cephalothrix hongkongiensis, Cephalothrix linearis*, and *Tubulanus polymorphus*) and Hoplonemertea (*Malacobdella grossa* and *Paranemertes peregrina*). However, the only transcripts identified representing known nemertean-specific toxin candidates were those resembling cytotoxin A-III in *C. marginatus*, *L. longissimus*, *L. ruber*, and *L. lacteus* [18]. Interestingly, the cytotoxin A-III family also contains the only nemertean-specific toxin class, which was identified in the first nemertean whole genome sequence (*Notospermus geniculatus*) and appears to have undergone evolutionary expansion [3].

Predicting toxins based on genome or transcriptome data is unreliable without corresponding proteome data because transcriptome-only data in particular increase the number of false positives and therefore overestimates putative toxin matches [19,20]. However, integrated proteomic and genomic/transcriptomic data (proteogenomics and proteotranscriptomics) are not yet available for nemertean species. Here we present the first proteotranscriptomic analysis of putative toxins from the hoplonemertean *A. lactifloreus* that inhabits lower shores—for example, under rocks or stones across European costs—and was collected in the tidal zone of the German Wadden Sea on Sylt. Samples of proteins secreted from the proboscis (PrS), epidermis (EpS), and entire mucus layer (MuS) were compared to the proboscis-specific transcriptome (posterior and anterior proboscis, including the stylet apparatus) to assess the abundance of toxin-related transcripts and corresponding proteins, focusing on the proboscis as the main structure potentially used for toxin secretion.

## 2. Results

### 2.1. No Nemertean-Specific Toxin Transcripts Are Present in the A. lactifloreus Proboscis Transcriptome

We analyzed putative toxin sequences in the *A. lactifloreus* proboscis transcriptome (Table 1). These were inferred from 41,377 open reading frames (ORFs) predicted with Transdecoder v5.0.2 [21] based on 96,851 transcripts that were assembled with Trinity v2.8.4 [22]. A BLASTP search (Basic local alignment search tool with a relaxed e-value of ≤0.001) was used to screen a manually curated database of all known nemertean-specific toxins (Additional File 1) against the *A. lactifloreus* proboscis transcripts. Additionally, the only published nemertean proboscis transcriptome (*N. geniculatus* [3]), which was assembled and processed in the same manner as the transcriptome of *A. lactifloreus*, was analyzed in parallel to test whether nemertean-specific toxins are generally expressed in the proboscis.

The *A. lactifloreus* proboscis transcriptome did not contain any transcripts with significant similarity to the known nemertean-specific toxin groups: nemertide α, neurotoxins B-II and B-IV, parbolysin or cytoxoin A-III. However, we found sequences similar to *N. geniculatus* nemertides α-1 and α-2 and parbolysin (Additional Files 2 and 3) that were not identified in the original study on *N. genicularis* [3]. Interestingly, cytotoxin A-III was not found in the *N. geniculatus* proboscis transcriptome, despite the presence of a corresponding gene in the recently published *N. geniculatus* genome assembly, where it appears to have expanded and is predominantly expressed in egg tissue [3]. We were unable to obtain sufficient protein for mass spectrometry from *A. lactifloreus* proboscis samples and it was, therefore, not possible to validate proboscis-expressed transcripts against the corresponding proteomic data.

### 2.2. Metalloproteinase M12 and Actitoxin-Like Transcripts Are the Most Abundant Putative Toxin Transcripts in the Proboscis of A. lactifloreus

In addition to nemertean-specific toxins, we mined the *A. lactifloreus* proboscis transcriptome for toxin proteins identified in other animals. To reduce false-positive matches, we compared the 1335 transcripts from the resulting BLASTP search against the ToxProt database of known toxins (e ≤ 0.001) versus normal physiological variants from SwissProt (e ≤ 0.001). Several hits against known toxins were identified as matches (Table S1). These were stringently filtered to exclude false-positives [19,20]. We only accepted transcripts (1) with similar or higher e-values and bitscore values in their ToxProt annotation compared to the SwissProt matches and (2) that were validated in comparative alignments with the corresponding toxin and, if available, non-toxin sequences. We also applied a threshold minimum expression level of two transcripts per million (≥TPM 2).

**Figure 2:**
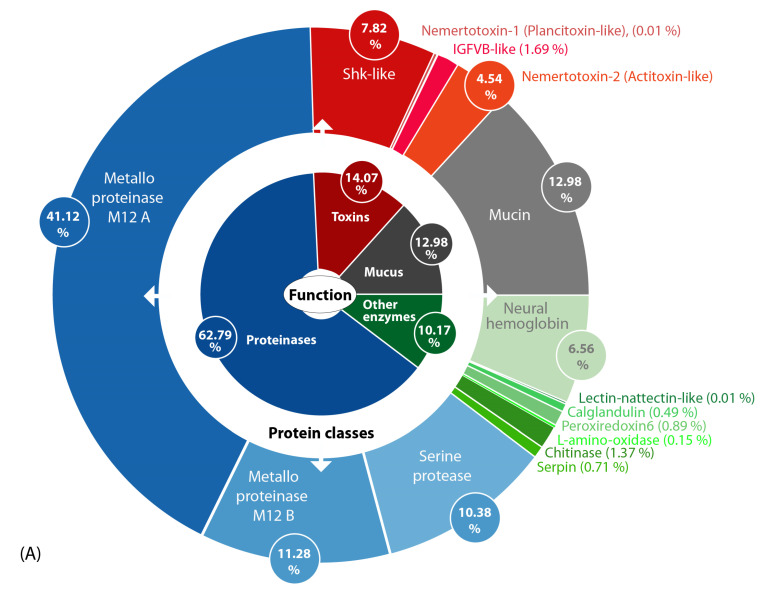

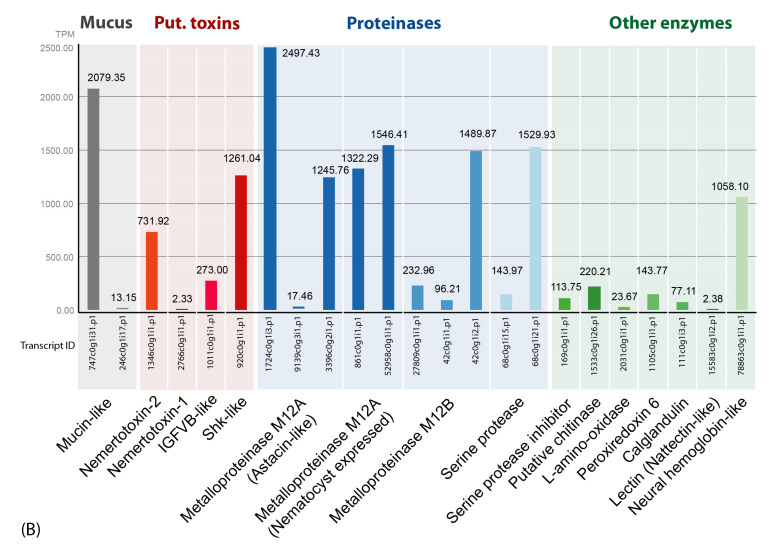
(**A**) Overview of toxin composition and identified putative toxin protein classes (outer circle) in the skin and mucus secretions of *A. lactifloreus* according to biological function (inner circle). The values reflect the percentage TPM (transcripts per million) values of transcripts grouped in the corresponding protein classes. (**B**) Bar chart showing the expression level for each transcript in TPM values (only transcripts identified by Mascot-based analysis (Table S2) are included).

Four major toxin-related groups were present in the *A. lactifloreus* proboscis transcriptome (Table 1). The first and most abundant group comprised proteinases, including metalloproteinase M12 candidates with particularly high expression levels. The second group included actitoxin-like transcripts similar to short cnidarian actitoxins, which feature a cysteine-rich Kunitz-BPTI domain, and larger plancitoxin-like transcripts identified in other nemertean transcriptomes [2,18]. This group also included transcripts related to three clades of the inhibitor cystine knot (ICK)/knottin family, specifically conotoxin-like proteins featuring 4-C scaffolds (putative R-superfamily conotoxins [23] known from Villepin’s cone, *Conus villepinii*), conotoxin-like proteins featuring 8-C scaffolds (similar to xibalbin-1 ICK sequences in remipede and several spider-like venoms [24,25]) and ICK-like calcium channel inhibitors from scorpions. The actitoxin-like transcripts were the most abundant, followed by the plancitoxin-like and ICK-like transcripts. The third group comprised non-protease enzymes, including galactose-specific lectin and calglandulin transcripts that were particularly abundant. Finally, the fourth group accounted for the remaining proteins, including several growth factor-like transcripts with low expression levels. A subset of protein families expressed in the proboscis transcriptome was also identified in the mucus proteome, which supports our findings concerning the nemertean-specific toxins of *N. geniculatus* (Table 1 and Figure 2).

### 2.3. Proteinases Dominate the Skin and Mucus Secretions Accompanied by Mucins, Enzymes and Putative Toxins

The epidermal secreted proteome (EpS) contained mostly degraded proteins, with two exceptions, making further characterization difficult (Table S1, see Additional File 4 for complete Mascot output). Most of our protein data were therefore derived from the mucus secreted proteome (MuS). Some of the nemertean-specific toxins identified in the mucus were also clearly expressed in the proboscis. We therefore used the proboscis tissue as a specific database and SwissProt as a nonspecific database for the proteome search. Complementary *A. lactifloreus* body transcriptomes could not be prepared.

Recent studies have shown that the exclusive analysis of proteome-supported transcripts can avoid the over-interpretation of false-positive hits from transcriptome-only data [19,20]. We therefore restricted our downstream analysis to the transcripts also represented in the proteome data (Table S2). Furthermore, we only included transcripts identified in the EpS and MuS proteomic datasets with Mascot values ≥ 24 based on at least two matching peptides (Table S2; see Additional Files 4 and 5 for complete Mascot output). We therefore retrieved 314 transcripts with predicted ORFs that survived these stringent filtering criteria (Table S2). Full annotation tables and corresponding sequences for all predicted ORFs using BLASTP (e ≤ 0.001) against nemertean-specific toxins, SwissProt, ToxProt, antimicrobial peptides, and Interpro scan v5 [26] are provided in the additional data.

Many toxins arise from the duplication of genes encoding proteins and peptides with normal physiological functions, resulting in high similarities between the neofunctionalized toxin and its ancestral protein [17,27,28]. To reduce false-positive matches, we compared the BLASTP results of the 314 candidate transcripts (e ≤ 0.001) against nemertean-specific toxins, known toxins (ToxProt), and known proteins including normal physiological variants (SwissProt). We accepted the transcripts as putative toxins only if the bitscore values of the nemertean toxin or ToxProt-derived transcripts were similar or higher than the SwissProt matches. The final 23 transcripts surviving this selection process represented 15 protein families in four major categories with different biological functions and expression levels (Figure 2).

The putative toxin cocktail defined by transcriptome data and refined by proteomic validation is dominated by a primary group of proteinase transcripts, particularly those encoding M12A and M12B metalloproteinases and serine proteinases. The second group of transcripts encodes other enzymes, including abundant chitinase and serpin transcripts (Figure 2). The third group encodes mucus-related proteins (mucins) that have also been found in amphibian skin secretions [29]. The fourth group encodes four toxin protein families: (1) IGFBP-like proteins (insulin-like growth factor binding proteins), which are found in the venoms of invertebrates, such as scorpions and remipedes [24,30]; (2) plancitoxins, previously identified in the crown-of-thorns starfish, a venomous echinoderm [31,32]; (3) actitoxins, previously identified in sea anemones [33]; (4) ShK-like proteins (*Stichodactyla*-like proteins) that are also major venom components in sea anemones [34]. The expression levels of each transcript are presented in relative values as a pie chart (Figure 2A) and also in absolute TPM values as a bar chart (Figure 2B). Interestingly, several representatives of protein families that were identified in the proboscis transcriptome also appear to be secreted into the mucus proteome. However, some of the more abundant transcripts from these same families are not present in the mucus proteome (Figure 2; Table 1 and Table S1).

**Figure 3:**
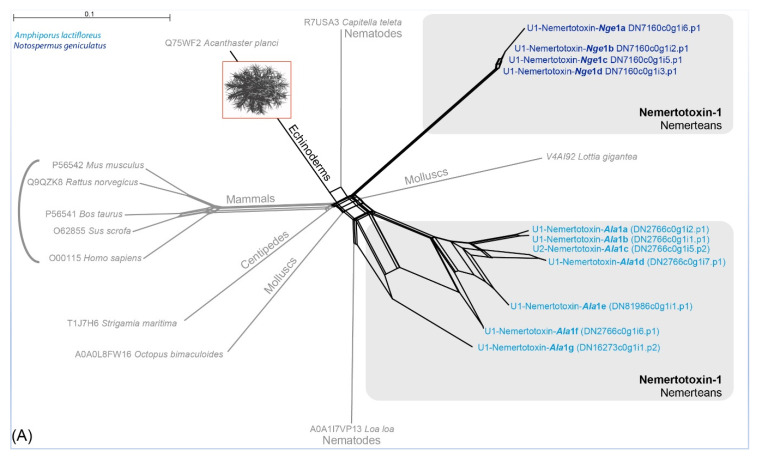

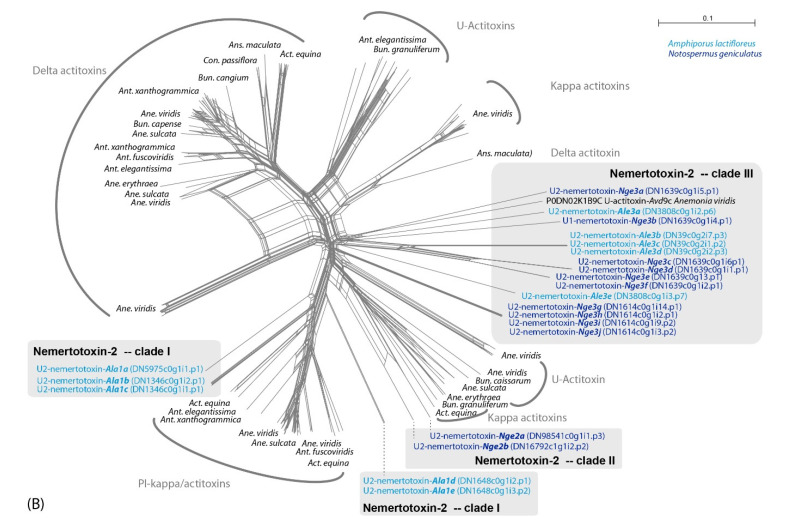
Neighbor-joining network reconstruction of all identified nemertotoxin-1 (**A**) and nemertotoxin-2 (**B**) sequences in Splitstree 5 [37] with standard settings based on the uncorrected *p*-distances. *A. lactifloreus* and *N. geniculatus* sequences are shown in light and dark blue, respectively. Genus abbreviations for sea anemone taxa: Act = *Actinia*, Ane = *Anemonia*, Ant = *Anthopleura*, Ans = *Antheopsis*, Bun = *Bundosoma*, Con = *Condylactis*.

Our results support a hypothesis in which the identified actitoxin-like and plancitoxin-like proteins resemble two potential nemertean-typical families in the toxin arsenal of this phylum. Variants of both toxin families were also detected in the proboscis transcriptome of *N. geniculatus* and in the full-body transcriptome assemblies of seven other species from all three nemertean clades [2,18]. We name these new putative toxin classes U-nemertotoxin-1 and U-nemertotoxin-2, following the convention of Undheim and colleagues [35,36]. The *N. geniculatus* and *A. lactifloreus* U-nemertotoxin-1 variants are distinct, separating into different clades (Figure 3A). Furthermore, U-nemertotoxin-2 appears to have diversified into three different clades: clade I is similar to PI actitoxins from sea anemones and consists only of *A. lactifloreus* sequences; clade II consists only of the *N. geniculatus*; clade III features sequences from both nemerteans, most similar to U-actitoxins from the sea anemone *Actinia viridis* (Figure 3B).

### 2.4. Several Secreted Proteins Are Strongly Expressed but Remain Mostly Uncharacterized

Several of the proteins we identified were strongly expressed but only rudimentary annotations are available from InterPro scan (e.g., indicating whether or not a signal peptide or non-cytoplasmic domain is present). This group included the most abundant transcript in the proboscis transcriptome (DN187_c0_g1_i14.p1). None of these sequences generated hits in the NCBI non-redunant database. We do not discuss these matches further, given the lack of additional information, but it is possible that some of them represent novel toxin proteins (Table 2).

### 2.5. No Putative Antimicrobial Peptides Were Identified in the A. lactifloreus Proteotranscriptome

Marine organisms often produce strong antimicrobial secretions on the epidermis as protection against pathogens, so nemerteans offer a novel source of potential antimicrobial peptides [38,39]. Given that *A. lactifloreus* is a littoral species and probably more exposed to microbial organisms, we used BLASTP (e ≤ 0.001) to screen our proteome data against the antimicrobial peptide database 3 (APD 3) [40]. We did not find any matches, which suggests that *A. lactifloreus* does not secrete any proteins or peptides that are similar to known antimicrobial peptides.

## 3. Discussion

### 3.1. Are Known Nemertean Toxins Taxon-Specific?

**Figure 4:**
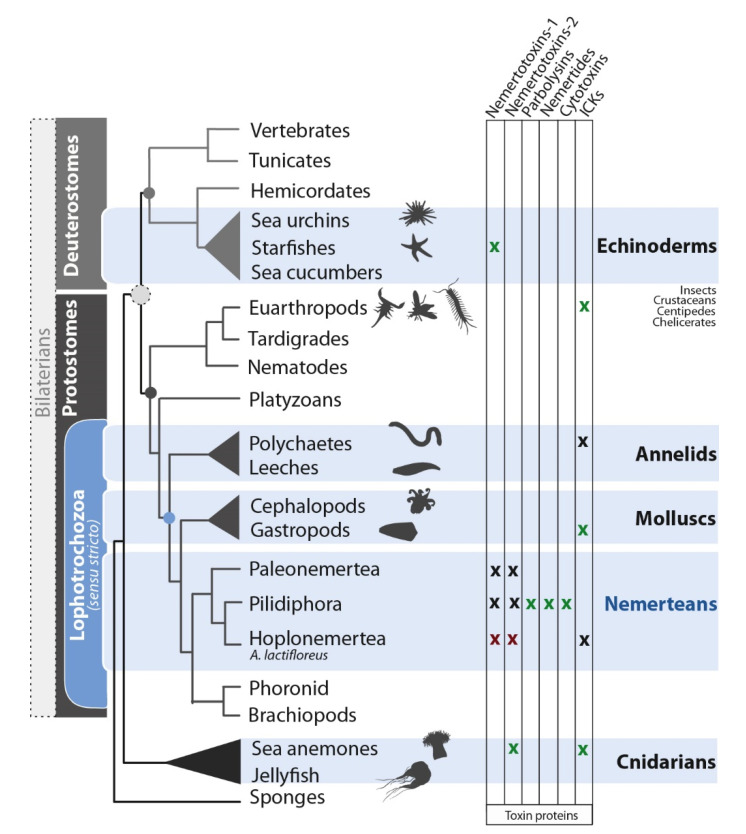
A cladogram illustrating the relationships among venomous, marine animal lineages (emphasis on nemerteans), showing the distribution of toxin protein classes. A green x indicates a known characterized toxin, a red x indicates proteotranscriptomic matches from *A. lactifloreus*, and a black x indicates toxin candidates supported by transcriptome data alone. The phylogeny is based on a recent genomics analysis [3]. Some lineages have been pruned for simplicity.

Nemerteans (ribbon worms) use toxins for predation and defense, similar to annelids and mollusks (also representing the superphylum Lophotrochozoa) and more distant phyla, such as echinoderms and cnidarians (Figure 4). Research on nemertean toxins has focused on small-molecule compounds in the mucosal secretions, whereas little is known about larger toxin proteins secreted by the epidermis and proboscis. We addressed this issue by comparing the proboscis transcriptome and mucus proteome of *A. lactifloreus* in order to cross-validate the toxin-related transcripts we identified. Our proteotranscriptomic analysis revealed no variants of known nemertean-specific toxin proteins (neurotoxins, cytotoxins, parbolysins, or nemertides), which were discovered in three non-hoplonemertean species: *C. lacteus*, *P. corrugatus*, and *L. longissimus* (Figure 4). These proteins may therefore be restricted to particular species, genera, or other taxa, rather than representing typical nemertean toxins. Interestingly, expressed variants of two of the described toxins (parbolysin and both nemertides) are present in the proboscis transcriptome of *N. geniculatus* (class Pilidiophora, order Heteronemertea), while they lack in the proboscis transcriptome of *A. lactifloreus*. Hoplonemerteans, such as *A. lactifloreus*, have probably recruited toxins that differ from those identified in the limited range of nemertean species sampled thus far. Potential new neurotoxin candidates include the three distinct groups of ICKs in the *A. lactifloreus* proboscis transcriptome, similar to those isolated from cones (*C. villepinii*), remipedes/spiders, and scorpions, respectively. The *A. lactifloreus* sequences are new variants that provide insight into the evolutionary diversity of ICKs among different invertebrate taxa. However, given the lack of complementary proteome data and the low abundance of transcripts, we do not discuss these candidates further.

We introduce here two new putative nemertean toxin classes, U-nemertotoxin-1 and U-nemertotoxin-2, noting that the naming of toxins in general has yet to be standardized. It is likely that future research will identify additional sequences of known nemertean toxins and of new (untested and putative) candidates based on extended taxon sampling, so the ultimate naming convention for nemertean toxins should include evolutionary/systematic and biochemical aspects to cover these candidates, better integrating the phylogenetic context.

### 3.2. The Putative Venom Cocktail of A. lactifloreus and Its Mode of Action

Predatory nemerteans attack with fast strikes and return later to consume their incapacitated prey, which is consistent with the delivery of known paralytic neurotoxins [6,7]. Although we identified none of the known nemertean toxin families in *A. lactifloreus*, it is likely that nemertotoxins 1 and 2 act in a similar manner. The predator attacks, pierces the prey with the stylet, introduces the cocktail of toxins, and waits for the prey to become immobile before consumption. We propose that the mixture of proteinases and other enzymes supports the activity of the neurotoxins and also begins the process of pre-digestion, as reported for many other venomous taxa, including assassin bugs, robber flies, and remipedes [24,41,42]. Pre-digestion is necessary because *A. lactifloreus* belongs to the order Monostillifera (nemerteans with only one stylet), which feeds by sucking liquefied prey tissues through the joint opening of the mouth and proboscis pore.

Several of the proteins expressed in the proboscis of *A. lactifloreus* are also secreted in the mucus. We speculate that proteins secreted in the mucus facilitate predation and the paralysis of prey by exacerbating the wound so that neurotoxic components reach their target more rapidly, a mechanism deployed by some amphibians [43]. The nemertean proboscis evolved as a venom apparatus by recruiting toxic proteins from the epidermal glands into proboscis-specific cells, supporting a hypothesis in which the proboscis outer epithelium is developmentally analogous to an inverted part of the epidermis. The stylet apparatus (one stylet in *A. lactifloreus* but multiple stylets in some other nemerteans) enables hoplonemerteans to mechanically rupture the prey’s skin and deliver the venom cocktail more efficiently. We therefore argue that hoplonemerteans possess a classic venom system and venom apparatus that allows them to penetrate their prey and inject a toxic cocktail [16,17].

### 3.3. General Use of Toxin Proteins and Their Mode of Action in Nemerteans

**Figure 5:**
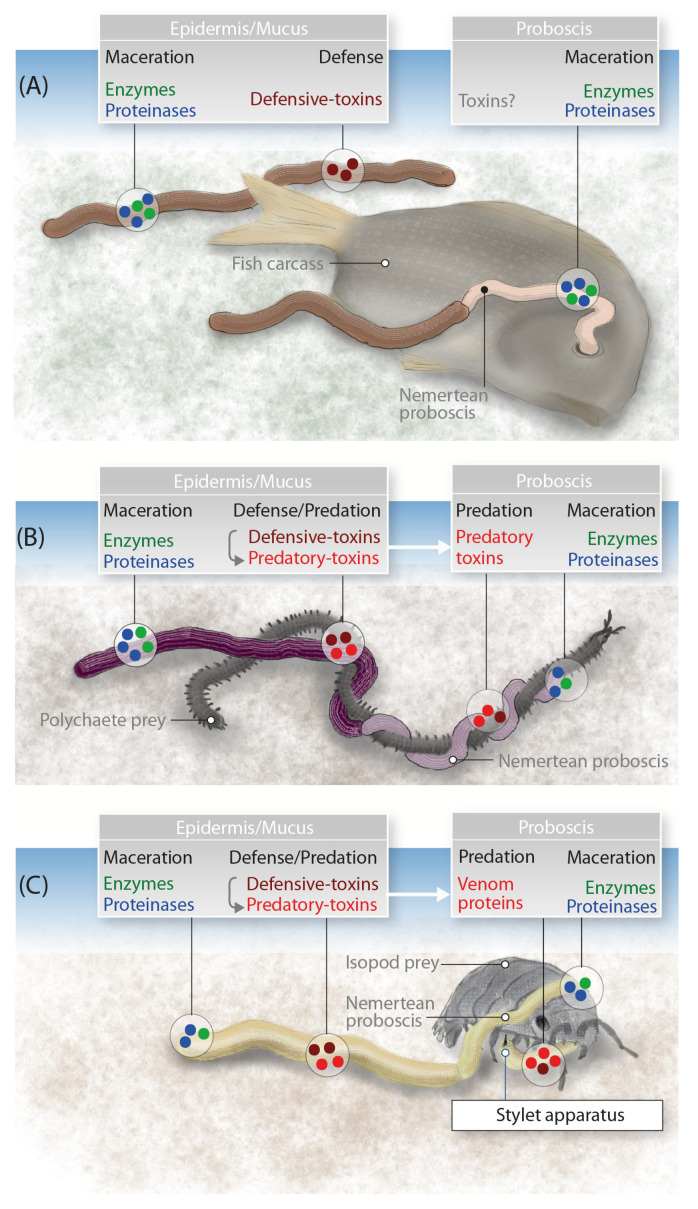
Model showing the evolutionary adaptations of toxin proteins and their expression in nemerteans with different feeding strategies. (**A**) A nemertean scavenger feeds on a fish carcass using the cocktail of proteases and other enzymes to macerate the tissue before ingestion, while toxins in the epidermis are used for defense. (**B**) A pilidiophoran that lacks a stylet apparatus captures a polychaete using paralytic toxins recruited into the proboscis. The proteases and other enzymes help the toxins reach their targets rapidly, and also facilitate a pre-digestion. (**C**) A hoplonemertean, such as *A. lactifloreus*, uses its venom apparatus to overpower an isopod crustacean with its prey-piercing stylet and toxins expressed in the proboscis.

The toxins produced by nemerteans occupy a grey area in terms of definition, because some (predatory) species deploy them as venoms whereas others (scavengers) use them solely for passive defense and pre-digestion. One example of the latter is *P. corrugatus*, which uses its secretions to melt its way into prey flesh and gain access to internal tissues, facilitated by secreted proteinases and other enzymes (Figure 5A). In more recent evolutionary history, the proteins in the mucus (and possibly in the proboscis) have gained the ability to paralyze and kill prey enveloped by predatory nemertean species. In such cases, the secreted proteinases and other enzymes are initially used to rupture the prey and promote the activity of the toxins, at the same time gaining access to the body cavity for pre-digestion before ingestion, similar to the role in scavenging species (Figure 5B). The efficiency of these enzymes makes them attractive targets for applied research. Hoplonemerteans mastered this predatory strategy by evolving a piercing structure to deploy their venom cocktail more efficiently, which might be accompanied by different toxins that evolved in parallel with the stylet apparatus.

## 4. Conclusions

Nemertean secreted protein toxins have received little attention from the research community. We carried out the first proteotranscriptomic analysis of a species from the hoplonemertean lineage, leading to a model of toxin evolution coincident with the transition of nemerteans from scavengers to predators. A more comprehensive understanding of nemertean toxins will require the comparison of diverse species representing the entire phylum, studies that consider the role of nemertean mucus as a mechanically protective, toxin-carrying matrix, and an analysis of the proboscis as a structure for defense and predation. Of particular interest are spatial differences in the expression and secretion of proteins by the epidermis and proboscis. Hoplonemerteans provide an ideal model to characterize the transition from defensive toxins to predatory toxins in combination with the evolutionary adaptation of a distinct venom apparatus. The venom secreted by nemerteans also offers a potentially valuable source of novel enzymes and antimicrobial proteins.

## 5. Materials and Methods

### 5.1. Collection and Preparation of A. lactifloreus Specimens

**Figure 6:**
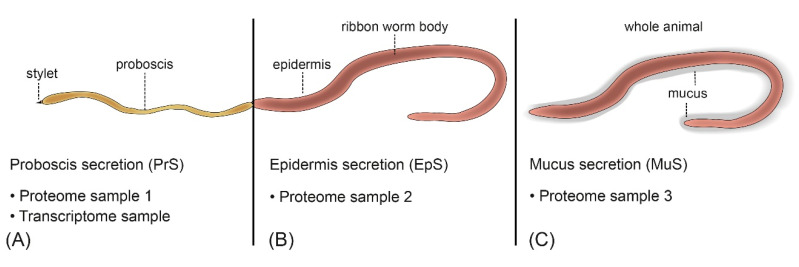
Sampling strategy for the proteotranscriptomic analysis of *A. lactifloreus* shown with an everted proboscis. (**A**) The whole proboscis was used to scrape off secreted proteins and ultimately to generate transcriptome data. (**B**) The epidermis was sampled for secreted proteins. (**C**) The mucus around the whole animal was taken for proteome sample 3.

Four *A. lactifloreus* specimens were collected in August 2019 from the tidal zone of the Wadden Sea near the Wadden Sea Station of the Alfred-Wegener Institute in List on the island Sylt, Germany. For proteomics analysis, each specimen was briefly washed in sterile phosphate-buffered saline (PBS) and agitated in a Petri dish with sterile salt water to induce evertion of the proboscis. This was transferred to a second Petri dish and scraped with a modified pipette tip to collect secreted proteins (PrS). The epidermis of each specimen was also scraped (EpS). Three minutes later, the mucus was scraped from the entire body (MuS). Each sample of secreted protein was separately deposited in UltrPure distilled water (Thermo Fisher Scientific, Waltham, MA, USA) and the corresponding samples from all four individuals were pooled and stored at −80 °C for further analysis (Figure 6). The proboscis (including the anterior and posterior parts, and stylet apparatus) was dissected in sterile PBS and immediately macerated and stored in RNAlater (Thermo Fisher Scientific) at −20 °C for transcriptome sequencing.

### 5.2. RNA Isolation, Library Preparation and Illumina Sequencing

RNA extraction and sequencing were outsourced to Macrogen, Seoul, Korea. After total RNA extraction using a low-input protocol, RNA quality and integrity were tested using a Bioanalyzer 2100 (Agilent Technologies, Santa Clara, CA, USA). The cDNA library was constructed using the TruSeq Stranded mRNA LT sample kit (Illumina, San Diego, CA, USA) for sequencing on an Ilumina HiSeq 2500 platform, generating 150-bp paired-end reads. In total 46,559,252 reads were generated with a GC% of 48.72. The quality score ratio for sequenced bases was 98.53 for phred 20 and 94.53 for phred 30.

### 5.3. Transcriptome Assembly, ORF Prediction and Identification of Venom Proteins

The raw sequencing reads were processed using the Animal Venomics Group in-house assembly and annotation pipeline. The reads were inspected using FastQC v.0.11.7 [44] and trimmed using Trimmomatic v0.38 [45] with standard settings, except the differing parameters—SLIDINGWINDOW: 4:30 MINLEN: 70. Additionally, a manually curated adapter file, which includes all possible sequencing adapters, was used instead of the provided adapter sequences (Additional File 6). All trimmed reads were then assembled in Trinity v2.8.4 [21,22] with standard settings, except a modified minimum contig length of 70 bp. Expression levels were quantified with Kallisto 0.46.1 [46] using standard settings. In parallel to the proboscis transcriptome of *A. lactifloreus*, the published proboscis transcriptome of *N. geniculatus* (SRR5811996) was processed using the same settings. The resulting assembly files are provided in Additional Files 7 and 8.

For all assembled transcripts, the ORFs were predicted and annotated with Transdecoder v5.0.2 [21] at the amino acid level, running Interproscan v5.27.6 [26] and BLASTX searches against the NCBI NR database (e ≤ 10^−6^). All ORFs predicted by Transdecoder (Additional Files 9 and 10) were analyzed to identify possible toxin peptides or proteins by screening against four specific protein databases using BLASTP (best match only, e ≤ 10^−6^): SwissProt with 561,690 entries, ToxProt with 7133 entries, NemerteanToxins (a manually curated, in-house nemertean-specific database) with 64 described toxins/venom proteins from ribbon worms, and the antimicrobial peptide database APD 3 [40] with 2338 activity-tested antimicrobial peptides. All database files used for BLAST searches are provided as Additional Files 1, 11–13 (all databases were accessed and downloaded for local searches on 15.03.2020). All BLASTP search results are provided in Additional Files 14–21.

The toxin candidate sequences were identified using BLASTP and subsequently aligned with known toxin sequences and, where available, with non-toxin variants to exclude false-positive matches using the mafft-LINSI algorithm [47]. All alignments of putative toxin protein classes are provided in Additional Files 22–23. All relevant information concerning the transcriptome data is accessible via the NCBI Bioproject PRJNA636261 and the SRA entry SRR11906528.

### 5.4. Peptide and Protein Identification

10 µg of protein were dissolved in 25 mM ammonium bicarbonate with 0.1 nM ProteasMax. Cysteine residues were reduced with 5 mM dithiothreitrol for 30 min at 50 °C and then modified for 30 min at 24 °C with 10 mM iodacetamide. The reaction was leveled with excess cysteine before adding 0.025 ng/µL trypsin in a total volume of 100 µL. The reaction was stopped by adding trifluoroacetic acid to a final concentration of 1% after incubation at 37 °C for 16 h. The sample was purified with a C18-ZipTip (Merck-Millipore, Darmstadt, Germany), then dried under vacuum and re-dissolved in 10 µL 0.1% trifluoroacetic acid. 

1 µg of the sample was loaded onto a 50 cm µPAC C18 column (Pharma Fluidics, Ghent, Belgium) in 0.1% formic acid at 35 °C for analysis. Peptides were eluted in a 3–44% linear gradient of acetonitrile over 240 min and afterwards washed with 72% acetonitrile at a constant flow rate of 300 nL/min using a Thermo Fisher Scientific UltiMate 3000RSLCnano device (Thermo Fisher, Waltham, MA, USA). Eluted samples were injected via an Advion TriVersa NanoMate (Advion BioSciences, Harlow, UK) with a spray voltage of 1.5 kV and a source temperature at 250 °C into an Orbitrap Eclipse Tribrid MS (Thermo Fisher, Waltham, MA, USA) in positive ionization mode. Full mass spectrum scans were acquired every 3 s over a mass range of *m/z* 375–1500 with a resolution of 120,000 and auto-gain control set to standard with a maximum injection time of 50 ms applying data-independent acquisition mode. The most intense ions (charge state 2–7) above a threshold ion count of 50,000 were selected in each cycle with an isolation window of 1.6 *m/z* for higher-energy collisional dissociation at a normalized collision energy of 30%. Fragment ion spectra were gained in the linear ion trap with the scan rate set to rapid, a normal mass range, and a maximum injection time of 100 ms. After fragmentation, selected precursor ions were excluded for 15 s. Data were received using Xcalibur v4.3.73.11. (Thermo Fisher, Waltham, MA, USA) and analyzed with Proteome Discoverer v2.4.0.305 (Thermo Fisher, Waltham, MA, USA).

### 5.5. Matching Proteome and Transcriptome Data

Mascot v2.6.2 was used to search against the proboscis transcriptome using a precursor ion mass tolerance of 10 ppm. Cysteine carbamidomethylation was considered as a global modification, the oxidation of methionine was considered as a variable modification, and one missed cleavage site was allowed. Fragment ion mass tolerance was set to 0.8 Da for the linear ion trap MS ^2^ detection. The false discovery rate for peptide identification was limited to 0.01 using a decoy database. For subsequent analysis, only matches with a Mascot score > 24 and at least two verified peptides were included (Table S2). Candidates fitting these criteria were de-grouped to find all similar sequences or isoforms in the transcriptome that corresponded to groups within Mascot. The expression levels in TPM were combined for transcripts with several sequences representing one group (Table S2). The proteome raw data are made available via the ProteomeXchange server with the accession numbers PXD019867 and PXD019872.

## Figures and Tables

**Table 1:** The inferred protein families of toxin candidates from the *A. lactifloreus* proboscis transcriptome. The original taxon in which the family was identified is named and, if known, its activity is described. The numbers of transcripts that survived our filter criteria are given (Similar or higher e-values and bitscore values compared to SwissProt annotations and in alignments). Numbers in brackets indicate the overall number of transcripts annotated via ToxProt (Table S1). The expression levels in transcripts per million (TPM) are only provided for validated transcripts. Protein families in bold were also identified in the mucus proteome and asterisks (*) indicate families with at least one identical transcript in both the proboscis transcriptome and mucus proteome (Figure 2).

ToxProt Annotation	Protein Family, “Actual” Scaffold	Original Taxon Source	Presumed Activity	Transcripts Included/(All)	Expression TPMs (Sum)
Toxin Candidates					
Actitoxin-like* (Nemertotoxin 2)	Actitoxins, Kunitz-BPTI	Sea anemones	Possible neurotoxicity	10/(32)	1052.76
Delta-actitoxin-like	Actitoxins, Kunitz-BPTI	Sea anemones	Possible neurotoxicity	2/(2)	134.56
Conotoxin-like	Conotoxin-like ICK 4-C scaffold	Mollusks	Possible neurotoxicity	1/(3)	19.74
Conotoxin-like	Xibalbin1-like ICK 8-C scaffold	Mollusks	Possible neurotoxicity	2/(3)	19.74
Plancitoxin-like* (Nemertotoxin 1)	DNase II	Starfishes	Possible hepatotoxicity	7/(7)	29.17
Putative calcium channel toxin Tx758-like	ICK, knottin cysteine rich	Scorpions	Possible neurotoxicity	1/(1)	85.10
Kunitz-type U1 aranetoxin-like	Kunitz-BPTI, cysteine-rich	Spiders	Possible neurotoxicity	1/(1)	9.42
Proteinase Candidates					
Astacin-like metalloproteinase*	Metalloproteinase M12A	Diverse species	Proteinase activity	8/(52)	2597.43
**Nematocyst expressed protein 6**	Metalloproteinase M12A	Cnidarians	Proteinase activity	1/(36)	29.92
**Metalloproteinase zinc-disintegrin**	Metalloproteinase ADAM	Diverse species	Proteinase activity	1/(23)	0.07
Enzyme Candidates					
Venom allergen 5	CAP, Cysteine-rich	Diverse species	Enzymatic	1/(5)	1.76
C-type lectin (lectotoxin)	Lectins, C-type lectin	Diverse species	Enzymatic	3/(14)	5.5
Galactose-specific lectin* (nattectin)	Lectins, C-type lectin	Diverse species	Enzymatic	2/(11)	3.20
Calglandulin*	Calglandulins, EF-hand motif	Diverse species	Enzymatic	3/(75)	8.58
Cysteine-rich protein	Diverse, cysteine-rich	Diverse species	Enzymatic	2/(25)	16.89
Hyaluronidase (Conhyal-Cn1)	Hyaluronidase	Diverse species	Enzymatic	6/(9)	10.68
Kunitz-type serine proteinase inhibitor	Serpin	Diverse species	Enzymatic	3/(3)	20.81
Phospholipase A2	Phospholipase A2	Diverse species	Enzymatic	4/(6)	713.59
Snaclec bitiscetin	Lectins	Diverse species	Enzymatic	4/(5)	4.38
Snaclec coagulation factor	Lectins	Diverse species	Enzymatic	3/(4)	3.05
Snake venom metalloproteinase inhibitor	SVMP	Diverse species	Proteinase activity	3/(7)	8.15
Snake venom 5 nucleotidase	5-Nuclease	Snakes	Proteinase activity	3/(4)	15.43
Other Protein Candidates					
Vascular endothelial growth factor	Growth factors	Diverse species	Possible spreading factor	1/(2)	4.42
Venom nerve growth factor	Growth factors	Diverse species	Possible spreading factor	1/(2)	10.16
**Insulin-like growth factor (IGFVB)**	Growth factors	Diverse species	Possible spreading factor	3/(49)	3
Neuropeptide prohormone-4	Hormone precursor	Diverse species	Possible spreading factor	2/(7)	155.35

Asterisks (*) indicate families with at least one identical transcript in both the proboscis transcriptome and mucus proteome.

**Table 2:** Overview of the 29 novel proteins in the *A. lactifloreus* mucus proteome without detailed annotations. Transcripts without a signal peptide prediction are noted with NA (not applicable).

Transcript	TPM Value	Mascot Score	Length (aa)	Signal Peptide	Scaffold/Domain Prediction (Sequence Residue)
DN187_c0_g1_i14.p1	8524.05	195	161	yes (1–17)	Non-cytoplasmic domain (18–160), disorder prediction (137–160)
DN66444_c0_g1_i1.p1	2276.01	117	144	yes (1–19)	Non-cytoplasmic domain (20–144)
DN2243_c0_g1_i6.p1	2243.13	104	158	yes (1–22)	Non-cytoplasmic domain (23–158)
DN904_c0_g1_i1.p1	1328.59	177	100	yes (1–18)	Non-cytoplasmic domain (19–100)
DN416_c0_g1_i1.p1	1173.91	70	198	yes (1–19)	Non-cytoplasmic domain (20–198)
DN2192_c0_g1_i1.p1	709.61	114	117	yes (1–20)	Non-cytoplasmic domain (21–116)
DN4062_c0_g1_i1.p1	558.79	60	210	yes (1–23)	Non-cytoplasmic domain (24–210)
DN629_c0_g1_i1.p1	513.04	48	117	yes (1–43)	Non-cytoplasmic domain (44–117)
DN4200_c0_g1_i1.p1	394.49	101	122	yes (1–22)	Non-cytoplasmic domain (23–122), disorder prediction (20–108)
DN1209_c0_g2_i1.p1	143.95	62	309	yes (1–16)	Non cytoplasmic domain (17–308), disorder prediction (26–135), proline rich
DN5_c1_g1_i1.p1	140.00	78	117	no	Disorder prediction, coil (54–74)
DN355_c0_g1_i1.p1	36.43	30	107	yes (1–34)	Non-cytoplasmic domain (15–107), disorder prediction (38–65)
DN21169_c0_g1_i1.p1	6.28	95	120	yes (1–20)	Non-cytoplasmic domain (21–119)
DN5485_c1_g1_i2.p1	6.13	150	121	no	Galactose-like binding sf, unknown (11–120)
DN646_c1_g1_i1.p1	3.93	25	330	yes (1–20)	Non-cytoplasmic domain (21–330), disorder prediction (121–143), EGGSHELL
DN9918_c0_g1_i1.p1	3.40	239	204	yes (1–18)	Non-cytoplasmic domain (19203–), Unknown (25–177)
DN68091_c0_g1_i1.p1	3.28	137	121	no	Unknown unintegrated (41–113)
DN11320_c0_g1_i1.p1	2.83	280	113	yes (1–17)	Non-cytoplasmic domain (18–113)
DN70554_c0_g1_i1.p1	2.80	305	112	no	Disorder prediction (1–25)
DN39869_c0_g1_i1.p1	2.30	146	157	yes (1–17)	Non-cytoplasmic domain (18–157)
DN8497_c0_g1_i1.p2	2.16	60	131	yes (1–23)	Non-cytoplasmic domain (24–131)
DN16202_c0_g2_i1.p1	2.07	223	279	no	NA, proline and cysteine rich, disorder prediction (101–139)
DN40599_c0_g1_i1.p1	1.79	65	152	NA	NA
DN7825_c0_g1_i1.p1	1.66	176	223	no	Non-cytoplasmic domain (37–222)
DN16202_c0_g1_i1.p1	1.52	461	282	no	NA, proline and cysteine rich
DN40858_c0_g1_i1.p1	1.36	47	167	NA	NA
DN78568_c0_g1_i1.p1	1.28	99	146	no	Non-cytoplasmic domain (22–97)
DN60477_c0_g1_i1.p1	1.11	44	169	no	Unknown integrated (1-121), cysteine rich
DN7590_c0_g1_i1.p1	0.99	278	168	no	Unknown unintegrated (1–121)

## Supplementary Materials

The following are available online at https://www.mdpi.com/1660-3397/18/8/407/s1, Table S1: Annotation and expression levels of all transcripts that match against ToxProt. Table S2: Annotations and expression levels of all transcripts supported by the proteome analysis and based on the Mascot output. **Externally Hosted Supplementary Files:** Additional data are provided in the open access database ZENODO: https://zenodo.org/record/3908343 (doi:10.5281/zenodo.3908343). Additional file 1: BLASTP database of known nemertean toxins, Additional file 2: Alignment of matching nemertide sequences from *N. genicularis*, Additional file 3: Alignment of matching parbolysin sequences from *N. geniculatus*, Additional file 4: Mascot-based result table of the epidermis proteome sequences, Additional file 5: Mascot-based result table of the mucus proteome sequences, Additional file 6: Used Illumina adapter-file, Additional file 7: Assembly file of *A. lactifloreus*, Additional file 8: Assembly file of *N. geniculatus*, Additional file 9: Transdecoder file with predicted ORFs for *A. lactifloreus*, Additional file 10: Transdecoder file with predicted ORFs for *N. geniculatus*, Additional file 11: BLASTP database of SwissProt, Additional file 12: BLASTP database of ToxProt, Additional file 13: BLASTP database of antimicrobial peptides, Additional file 14: BLASTP results against known nemertean toxins for *A. lactifloreus*, Additional file 15: BLASTP results against known nemertean toxins for *N. geniculatus*, Additional file 16: BLASTP results against SwissProt for *A. lactifloreus*, Additional file 17: BLASTP results against SwissProt for *N. geniculatus*, Additional file 18: BLASTP results against ToxProt for *A. lactifloreus*, Additional file 19: BLASTP results against ToxProt for *N. genicularis*, Additional file 20: BLASTP results against AMP database for *A. lactifloreus*, Additional file 21: BLASTP results against AMP database for *N. geniculatus*, Additional file 22: Zip. file with all alignments of transcriptome-wise identified putative toxins; Additional file 23: Zip. file with all alignments of proteome-wise identified putative toxins.
